# Clinical Impact of Radiotherapy in Steroid-Refractory Graves' Orbitopathy

**DOI:** 10.7759/cureus.82981

**Published:** 2025-04-25

**Authors:** Elizabeth Escobar Peralta, José Manuel García Ramírez, Christian Helbert H Ordoñez, María Yisel Bautista Hernández

**Affiliations:** 1 Radiation Oncology, Cancer Center Tec 100, Querétaro, MEX; 2 Radiation Oncology, Hospital General de México "Dr. Eduardo Liceaga", Mexico City, MEX; 3 Radiation Oncology, Centro Regional de Radioterapia Zona Norte, Chihuahua, MEX

**Keywords:** exophthalmos, glucocorticoids, graves' orbitopathy, orbital radiation, radiotherapy, retrobulbar pain

## Abstract

Background: Graves' orbitopathy (GO) is the most common extrathyroidal manifestation of Graves' disease. Treatment options depend on its severity. Orbital radiotherapy is recommended as a second-line treatment in patients who are refractory or intolerant of glucocorticoid treatment. This study aimed to analyze the clinical outcome after radiotherapy in patients diagnosed with Graves' orbitopathy who are refractory to glucocorticoids.

Methods: We retrospectively analyzed the clinical data of 10 patients with Graves' orbitopathy who did not respond to glucocorticoid management. They were treated with retrobulbar radiotherapy between January 2010 and January 2021, with a schedule of 20 Gy in 10 fractions. The variables analyzed were ocular movements, exophthalmos, diplopia, retrobulbar pain, ectropion, visual acuity, eyelid closure, and conjunctival hyperemia.

Results: Categorical variables were compared before and after radiotherapy treatment in the evaluated patients. A statistically significant response to radiotherapy was observed in the following outcomes: exophthalmos (p=0.005) and retrobulbar pain (p=0.04). One case of local recurrence was observed at 20 months of follow-up, achieving a local control of 90%. Recurrence-free survival (RFS) at one, three, and five years was 100%, 90%, and 90%, respectively.

Conclusions: Radiotherapy is an effective and well-tolerated treatment that reduces exophthalmos and retrobulbar pain and can prevent disease progression.

## Introduction

Graves' orbitopathy (GO) is an inflammatory disorder of the orbit characterized by excessive glycosaminoglycan (GAG) deposition, an inflammatory infiltrate, and cytokine overproduction. It is the most common extrathyroidal disorder of Graves' disease, occurring in 30%-50% of these patients [1]. The reported incidence is 3-8/100,000 women per year and 1-1.5/100,000 men per year, with an overall prevalence of nine cases per 10,000 inhabitants [2]. GO has shown a bimodal peak: 40-44 years and 60-64 years in women, 45-49 years and 65-69 years in men [3].

Risk factors include age, gender, genetic factors, smoking with a 7.7% higher risk of developing GO (odds ratio (OR): 7.7, 95% confidence interval (CI): 4.3-13.7 versus OR: 1.9, 95% CI: 1.1-3.2) [4], thyroid dysfunction, radioactive iodine treatment, oxidative stress, selenium deficiency, intestinal flora imbalance, and hypercholesterolemia [5-8].

Orbital fibroblasts are the primary target cells in the autoimmune response. Upon activation by cytokines, these fibroblasts secrete large amounts of GAGs, predominantly hyaluronic acid, which expand the extracellular matrix surrounding the extraocular muscles. This process occurs without affecting the myofibrils. If this activated fibroblast is a pre-adipocyte, adipogenesis ensues, further increasing orbital fat volume. These changes contribute to elevated intraorbital pressure and impaired venous return, leading to proptosis and local inflammatory manifestations such as chemosis, periorbital edema, and, in severe cases, compressive optic neuropathy [9,10].

Treatment options for GO vary by severity and include high doses of glucocorticoids, selenium supplementation, and targeted therapies such as rituximab, teprotumumab, and tocilizumab. In moderate to severe cases (according to the European Group on Graves' Orbitopathy (EUGOGO) severity classification), surgery and orbital radiotherapy may be considered [11,12], especially in patients who are refractory or not candidates for glucocorticoids. Although retrospective studies have demonstrated the efficacy of orbital radiotherapy, results from prospective trials remain inconsistent [13-15]. Therefore, this study aimed to evaluate the clinical outcomes of orbital radiotherapy in patients with GO who were refractory to glucocorticoid therapy.

## Materials and methods

We retrospectively reviewed the clinical records of patients with a confirmed diagnosis of Graves' orbitopathy who were treated in the radiation oncology department at the General Hospital of Mexico between January 2010 and January 2021. Inclusion criteria were as follows: age > 18 years, treatment with radiotherapy using 3D conformal or intensity-modulated radiotherapy (IMRT) techniques, patients who received high doses of steroids without clinical improvement, complete medical records, and regular follow-up. Patients were excluded if they were lost to follow-up or had not received previous glucocorticoid treatment.

Thyroid hormone levels were not consistently documented and were therefore excluded from the analysis. It is also important to note that none of the patients received concurrent therapies such as selenium supplementation or other immunosuppressive agents. Furthermore, given their refractory response to high-dose glucocorticoids, no additional or low-dose steroid regimens were administered during the radiotherapy period.

Ethical approval was not requested for this study, as it involved a retrospective analysis of anonymized clinical data with no patient identifiers.

A total of 10 patients were obtained, eight (80%) women and two (20%) men, with a mean age at diagnosis of 43.4 years (28-62 years, standard deviation (SD): 12.7).

Patients were initially evaluated by an independent ophthalmologist not involved in the study, reducing observer bias. The clinical assessment included an ocular examination with visual field testing, and exophthalmos was measured using an ophthalmometer by determining the distance between the lateral orbital rim and the cornea. The EUGOGO severity classification was then applied. Mild GO presents one or more of the following: minor lid retraction (<2 mm), mild soft tissue involvement, exophthalmos < 3 mm above normal for race and gender, no or intermittent diplopia, and corneal exposure responsive to lubricants. Moderate to severe GO presents two or more of the following: lid retraction ≥ 2 mm, moderate or severe soft tissue involvement, exophthalmos ≥ 3 mm above normal for race and gender, and inconstant or constant diplopia.

The symptoms and signs at the beginning of radiotherapy of the 10 patients analyzed are shown in Table 1.

**Table 1:** Clinical manifestations before orbital radiotherapy

Signs and symptoms	Number (%)
Decreased eye movement	4 (40%)
Moderate exophthalmos	9 (90%)
Diplopia	5 (50%)
Retrobulbar pain	5 (50%)
Ectropion	1 (10%)
Decreased visual acuity	7 (70%)
Incomplete eyelid closure	5 (50%)
Conjunctival hyperemia	4 (40%)

**Figure 1:**
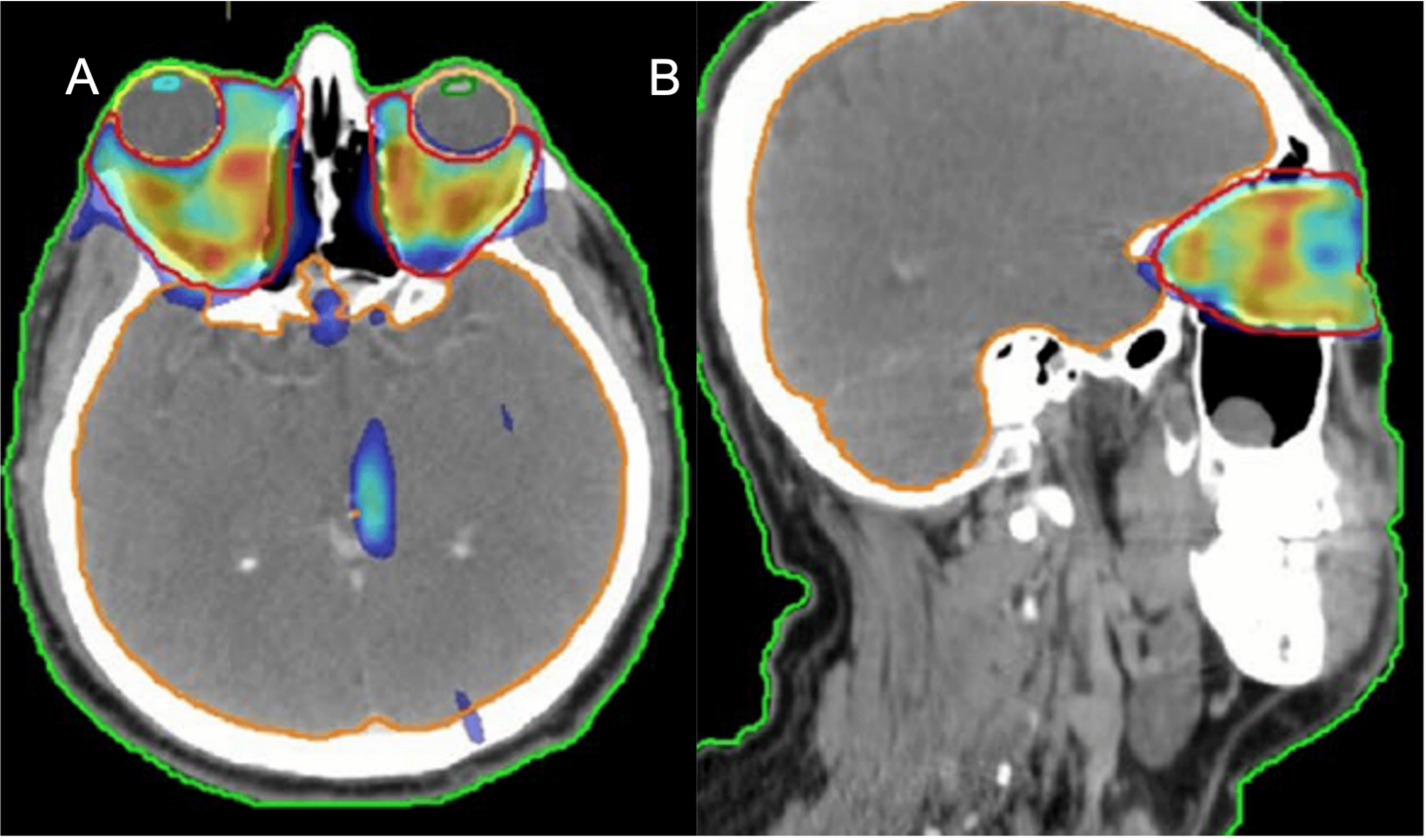
A 51-year-old woman diagnosed with Graves' orbitopathy underwent orbital radiotherapy using the IMRT technique, with a total dose of 20 Gy in 10 fractions a: Axial CT section showing the orbital structures and treatment planning. b: Sagittal section showing the dose distribution and target coverage. IMRT: intensity-modulated radiotherapy, CT: computed tomography

All patients were treated with retro-orbital irradiation using linear accelerator-based 3DCT (eight cases) or IMRT (two cases) techniques. Patients were immobilized with a custom-made thermoplastic case, and then a computed tomography (CT) scan without contrast was performed for image acquisition and target contouring. The clinical target volume (CTV) encompassed the origins to insertions of the extraocular muscles and the retro-orbital fatty spaces, with the main bulk. A total dose of 20 Gy was given to each patient in 10 fractions (Figure 1).

The median follow-up was 111 months (39-132 months, interquartile range (IQR): 50.3, 111, 118). The variables analyzed were ocular movements, exophthalmos, diplopia, retrobulbar pain, ectropion, visual acuity, eyelid closure, and conjunctival hyperemia. A paired comparative analysis of the variables was performed before and after radiotherapy treatment.

Statistical analysis

Categorical variables were described in frequencies and percentages. Numerical variables were described as means and standard deviations or medians with interquartile ranges, as appropriate. For univariate analysis, categorical variables were compared before and after radiotherapy treatment using the McNemar test. All statistical analyses were considered significant with a p-value < 0.05.

Recurrence-free survival (RFS) was defined as the time from the beginning of radiotherapy until disease recurrence. RFS was analyzed using the Kaplan-Meier method.

## Results

Categorical variables were compared before and after radiotherapy. No statistically significant differences were observed in eye movement (p=0.083), diplopia (p=1.0), ectropion (p=1.0), visual acuity (p=0.31), eyelid closure (p=0.08), or conjunctival hyperemia (p=0.54). In contrast, a statistically significant improvement was found in moderate exophthalmos, which decreased in 80% of patients, and retrobulbar pain, which resolved in 87.5% of affected patients. The significant outcomes are summarized in Table 2.

**Table 2:** Symptoms that improved after radiotherapy treatment (only positive findings are shown)

	Before treatment	After treatment	p-value
Moderate exophthalmos	8 (80%)	1 (10%)	0.005
Retrobulbar pain	5 (50%)	1 (10%)	0.04

Recurrence-free survival

**Figure 2:**
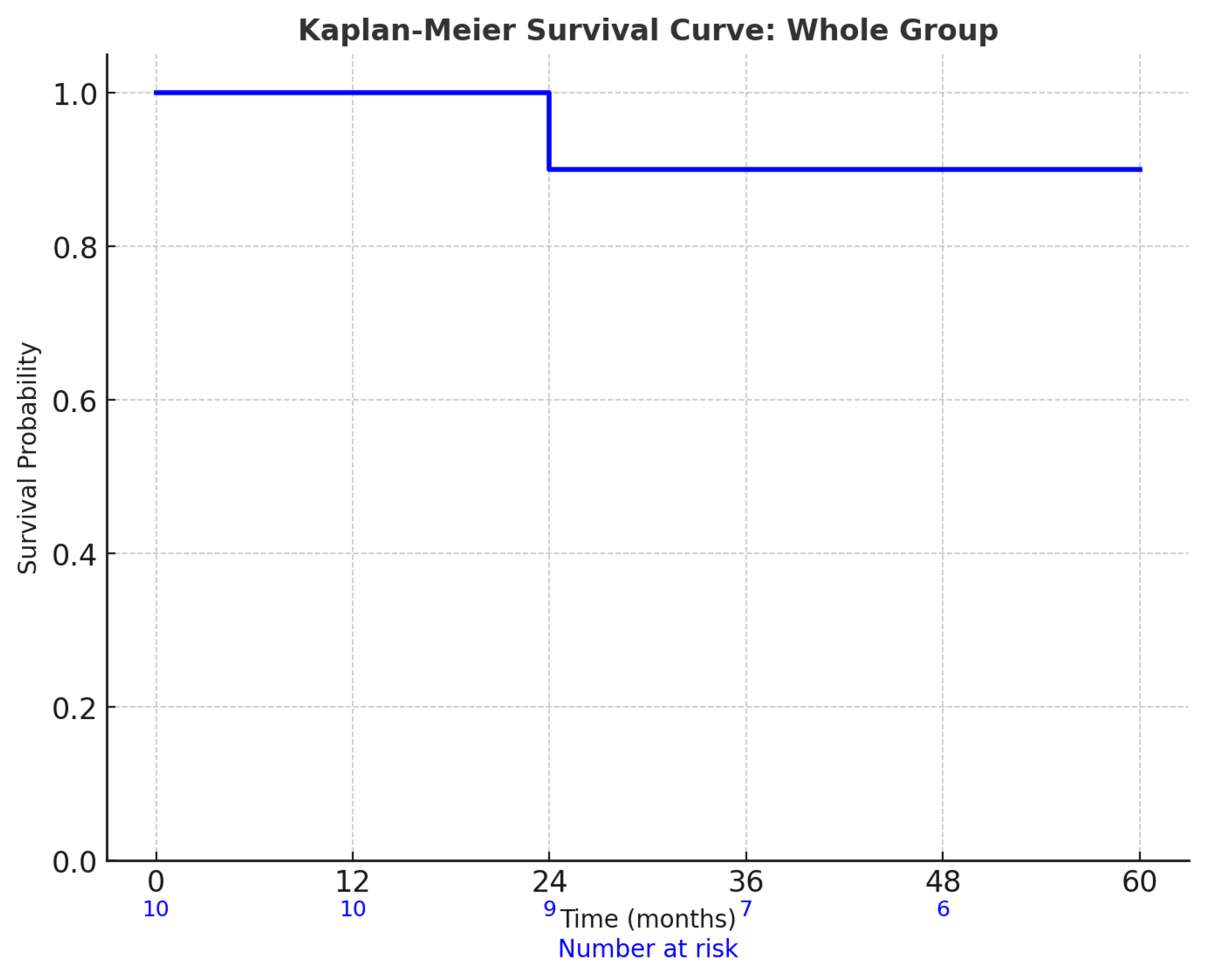
Kaplan-Meier survival curve: recurrence-free survival in orbital radiotherapy

One case of local recurrence was observed at 20 months of follow-up, resulting in an overall local control rate of 90%. Recurrence-free survival at one, three, and five years is detailed in Table 3, and the corresponding graph is shown in Figure 2.

**Table 3:** Recurrence-free survival CI: confidence interval

Time (months)	Recurrence-free survival	95% CI
12	100%	100-100
36	90%	73.2-100
60	90%	73.2-100

## Discussion

Several second-line treatment options have been proposed for patients with moderate to severe GO who do not respond to glucocorticoid therapy. These include tocilizumab, teprotumumab, and rituximab. These biologic agents have shown promising results but are limited by high cost, reduced accessibility, and potential adverse effects [16-18].

Radiation therapy for GO has been used for nearly a century [19]. Its anti-inflammatory effects can be observed at low doses (2.4 Gy), but higher doses are necessary to inhibit GAG synthesis by orbital fibroblasts. However, higher doses may also increase the risk of fibrosis in the extraocular muscles [20].

Several factors may influence the response to radiotherapy, including symptom duration (>12 months), male gender, hyperthyroidism, advanced age, diabetes, and smoking. The concurrent use of glucocorticoids can enhance the effectiveness of radiotherapy [21-23].

Radiotherapy is currently recommended as a second-line treatment for patients with moderate to severe GO who are either refractory to or have contraindications for glucocorticoids, providing sustained long-term efficacy [11,14,24].

A total dose of 20 Gy administered in 10 fractions (2 Gy/day) over two weeks is commonly used. Patients may experience a transient worsening of ocular symptoms, which can be controlled with low-dose oral prednisone [11].

Radiotherapy can be delivered using three-dimensional conformal radiotherapy (3D-CRT) with two opposite lateral fields or intensity-modulated radiotherapy (IMRT), which offers better conformity to the orbit, improves target coverage, and reduces toxicity to organs at risk such as the lens and optic nerves [25].

One limitation of this study is that eight patients were treated with the 3D technique (due to the treatment period), while only two received the IMRT technique. Consequently, a comparison between the two techniques cannot be made. Although orbital radiotherapy has previously demonstrated improvements in ocular mobility [26], our study found no statistically significant difference in this parameter (p=0.083). However, exophthalmos improved in 80% of patients (p=0.005), and retrobulbar pain resolved in 87.5% of symptomatic patients (p=0.04). No statistically significant improvements were observed in diplopia, visual acuity, ectropion, eyelid closure, or conjunctival hyperemia. Notably, none of these variables worsened during follow-up, suggesting that radiotherapy may help prevent disease progression.

In our series, overall disease control was achieved in 90% of patients, with a recurrence-free survival rate at one, three, and five years of 100%, 90%, and 90%, respectively. Based on these results, radiotherapy can be considered as a second-line treatment with acceptable toxicity.

## Conclusions

With the advent of glucocorticoids and targeted therapies, the management of patients with Graves' orbitopathy has improved. However, these treatments are often associated with considerable side effects. In this context, for patients either intolerant to glucocorticoids or refractory to this treatment, orbital radiotherapy with a dose of 20 Gy in 10 fractions offers a safe and effective alternative with durable clinical benefits.

Nonetheless, the present study has several limitations: the small number of patients, the absence of a control group, and the inability to compare outcomes between 3D-CRT and IMRT techniques. Future prospective studies with larger cohorts are warranted to validate and expand upon these results.
